# Readability of patient education materials on cardiac magnetic resonance imaging

**DOI:** 10.1093/ehjimp/qyaf111

**Published:** 2025-08-20

**Authors:** Albert Dayor Piersson, Bismark Ofori-Manteaw, Hanifatu Napari Mumuni, Klenam Dzefi-Tettey

**Affiliations:** School of Science, Technology & Health, Department of Diagnostic Radiography, York St John University, Lord Mayor’s Walk, York YO31 7EX, UK; Faculty of Science and Health, Charles Sturt University, Wagga Wagga, NSW 2678, Australia; Department of Statistical Sciences, Tamale Technical University, Tamale, Northern Region, Ghana; School of Medicine, Department of Radiology, University of Health and Allied Sciences, Ho, Volta Region, Ghana

**Keywords:** cardiac MRI, magnetic resonance imaging, readability, patient education materials, Flesch–Kincaid Reading ease

## Abstract

**Aims:**

We assessed the readability level of online patient education materials (PEMs) for cardiac MRI (CMRI) to determine whether they meet the standard health literacy needs as determined by the US National Institutes of Health and the American Medical Association guidelines.

**Methods and results:**

We evaluated the readability of CMRI PEMs from 5 websites using the Flesch–Kincaid Reading Ease (FKRE), Flesch–Kincaid grade level (FKGL), Gunning–Fog Index (GFI), Simple Measure of Gobbledygook index (SMOGI), Coleman–Liau Index (CLI), and Automated Readability Index (ARI). PEMs on the British Heart Foundation (BHF) website yielded the highest mean FKRE score, while the RadiologyInfo.org (RadInfo) website yielded the highest mean score on the CLI compared to all the other websites. Statistical analysis of individual predictors revealed that average words per sentence (*P* < 0.001) and average syllables per word (*P* < 0.001) were strong determinants of FKRE for the RadInfo PEMs. In contrast, sentences (*P* = 0.044), words (*P* = 0.046), average words per sentence (*P* = <0.001), and average syllables per word (*P* = <0.001) were significant predictors of FKRE for the InsideRadiology (InsRad) PEMs. The sensitivity analysis consistently confirmed the robustness and primary influence of average words per sentence and average syllables per word.

**Conclusion:**

The BHF and American Heart Association emphasize accessible CMRI communication, whereas RadInfo, InsRad, and the European Society of Cardiology PEMs may be less suitable for low-health-literacy audiences. Strategies aimed at enhancing the comprehensibility of patient education materials should primarily focus on reducing the average complexity of words and shortening average sentence lengths.

## Graphical Abstract

**Graphical Abstract:**
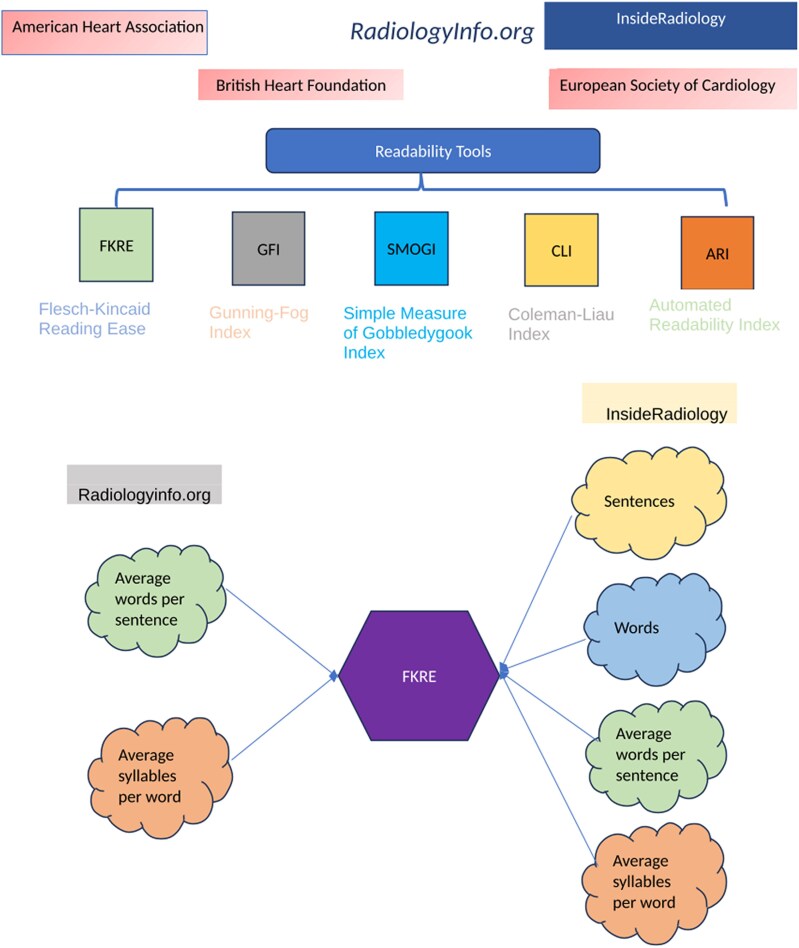
Assessment of readability level of online patient education materials on cardiac MRI.

## Introduction

Cardiovascular diseases (CVDs) remain the leading cause of mortality globally, estimated to take the lives of 17.9 million people each year, according to the World Health Organization.^1^ At the heart of CVD management is diagnostic imaging, which plays a crucial role not only in early detection but also in disease characterization and therapeutic planning. Among these important available imaging modalities, cardiovascular magnetic resonance imaging (CMRI) stands out as a non-ionising, non-invasive, and highly versatile technique that provides unparalleled structural, functional, and unique tissue characterization of the heart. Importantly, its ability to evaluate myocardial viability, perfusion, and fibrosis, in addition to its safety and accuracy, makes it the gold standard for diagnosing CVDs.^2,3^

However, the large size of the MRI scanner, the complex steps before and after a CMRI scan, and the use of unfamiliar medical terms can make the process confusing and stressful for patients, making it harder for them to understand the procedure and make informed decisions. In this regard, patient education materials (PEMs) represent an important interface between complex clinical procedures and the patients and relatives who may have to prepare for them. Unlike general radiological exams, CMRI often requires patient compliance with breath-holds, longer scan durations, contrast agent use, and specific pre-scan preparations—all of which necessitate clear, comprehensible, and condition-specific patient guidance. Moreover, CMRI is frequently used in the evaluation of complex or life-threatening cardiac conditions, meaning that any confusion caused by difficult-to-read PEMs may amplify health-related anxiety and impair informed consent. At most, it is crucial that practitioners understand patients’ anxiety as well as their information needs, fear of results, and coping strategies^4^ to foster trust and adherence to medical recommendations.

Despite identifying the importance of readability in PEMs, many materials fall short of recommended standards. For instance, a 20-year analysis of the readability of patient education materials (PEM) from high-impact medical journals^5^ found that only 2.1% of the materials met the American Medical Association recommendation of 6th-grade reading level, while 8.2% met the National Institutes of Health recommendation of 8th-grade level. According to the American Medical Association and American Medical Association Foundation, PEMs should be written at or below a 6th-grade reading level using active voice, one- or two-syllable words, short paragraphs, and simple tables and graphs.^6^ Active voice is usually more concise and puts the subject at the sentence beginning, providing the advantage of avoiding or cutting down excessive use of words.^7^

The average reading level of American adults is estimated to be between the 7th- and 8th-grade levels (12–14 years) according to the Literacy Project,^8^ and relatedly, the National Institutes of Health (NIH) advised that health materials be written at or below an 8th-grade reading level.^9^ Similarly, guidelines have been developed in the UK^10^ and other nations emphasizing simplicity and accessibility in health communication.

While resources on professional web portals are invaluable for disseminating important information for patients undergoing imaging, the readability and accessibility of PEM on CMRI remain largely underexplored. Existing literature on online PEM related to imaging has focused on different topics;^11–16^ however, several of them have pooled data across multiple imaging areas to deduce average readability, potentially masking insights into specific areas in imaging.

Our study tackles this gap by undertaking a focused analysis of the readability of CMRI PEMs from prominent organizations, and this includes the American Heart Association (AHA),^17^ British Heart Foundation (BHF),^18^  RadiologyInfo.org (RadInfo),^19^ InsideRadiology (InsRad),^20^ and the European Society of Cardiology (EsCardio),^21^ using robust readability scales. By so doing, we evaluated whether these PEM meet well-established standards and facilitate patient understanding of CMRI education. Identifying gaps coupled with proposing strategies to enhance improvement would contribute immensely to the broader discourse on health literacy and equitable access to healthcare information.

Research question: Do currently available online PEMs on CMRI from leading health organizations meet established readability standards for patient comprehension?

## Materials and methods

We evaluated the readability of PEMs on CMRI available on five prominent web portals: the AHA,^17^ the British Heart Foundation (BHF),^18^  RadiologyInfo.org (RadInfo),^19^ InsideRadiology (InsRad),^20^ and the European Society of Cardiology (EsCardio).^21^ These websites were selected based on their international reputation, credibility in cardiovascular education, and frequent citation in patient-facing and clinical resources.

The American Heart Association, founded in 1924, is the United States of America’s oldest and largest voluntary organization dedicated to fighting heart disease and stroke.^22^ The American Stroke Association, which is a division of the AHA, amplifies the efforts of the AHA to educate the public about stroke prevention and treatment. The AHA and American Stroke Association indicated they strive to make the websites accessible and that they are committed not only to diversity but also to inclusion, and meeting the needs of all their constituents, including those with disabilities.^23^ Further information on the website included their continuous improvement of digital assets to comply with the accessibility guidelines for levels A and AA in accordance with WCAG 2.1.^23^ We collated six questions/statements from the AHA website.

BHF is the United Kingdom’s biggest independent funder of heart and circulatory research; besides, it helps to find cures and treatments to give people more time with loved ones.^24^ The BHF website is owned and operated by or on behalf of BHF and aims to conform to level AA website accessibility standards of The World Wide Web Consortium (W3C) Web Content Accessibility Guidelines wherein it is indicated that the Web page satisfies all the Level A and Level AA Success Criteria, or a Level AA conforming alternate version is provided.^25^ Briefly, the intent of the guidelines is to make content accessible to a wide range of people with disabilities.^26^ We collated six questions/statements from the BHF website.

The Radiologyinfo was developed and sponsored by the American College of Radiology and the Radiological Society of North America. The website indicates that each section on its site was created through the guidance of a physician with expertise in the topic presented, with the aim of assuring their quality and accuracy.^27^ Further indication is that all information posted on the website is subject to further review by an RSNA-ACR committee, which is comprised of physicians with expertise in several radiologic areas.^27^ We collated 11 questions/statements from the RadInfo website.

The InsRad website is developed and maintained by the Royal Australian and New Zealand College of Radiologists.^28^ The website was designed to support the relationship that exists between a patient and his/her doctor.^28^ InsRad provides information items that have been written by radiologists or other health professionals who are experts in their field.^28^ The items have then been edited by a team of specialised consumer writers to ensure they have been made as easy to understand by health consumers, patients, and carers as possible.^28^ We collated 11 questions/statements from the InsRad website.

The European Society of Cardiology, which officially came into existence in 1950, was established to foster cardiology development and to further education in cardiovascular disease.^29^ We collated five questions/statements from the EsCardio website.

### Data collection and text extraction process

A comprehensive search was conducted of the indicated websites between the 15th and 24th of January, 2025. Inclusion criteria were patient educational materials written in the English language, publicly accessible, and focused on CMRI. Exclusion criteria included non-patient educational content, technical materials targeted at healthcare professionals, and materials that addressed non-cardiac imaging modalities.

For each site, the search function was used with terms such as ‘CMRI,’ ‘CMR,’ or ‘heart MRI’ to locate relevant PEMs intended for patient use. Only publicly accessible, English-language pages specifically explaining the procedure, indications, preparation, risks, and aftercare of CMRI were included. The full text from each page was copied directly into Microsoft Word document, and non-informative content—such as navigation menus, advertisements, author credentials, hyperlinks, and unrelated references—was removed. Texts were then formatted consistently to ensure uniformity across readability analyses.

### Readability assessment tools

The readability of the PEMs on the various portals was assessed using six different scales as indicated below. Multiple readability tools were used in our study to ensure a comprehensive and unbiased assessment of text complexity, as different algorithms apply distinct linguistic and statistical parameters, reducing the risk of methodological bias:

1. Flesch–Kincaid Reading Ease (FKRE), which typically ranges between 0 and 100. A high score indicates easier-to-read text while low scores indicate that the text is difficult to understand. The equation underlying FKRE is given by:
  206.835−1.015×(words/sentences)−84.6×(syllables/words)
2. Flesch–Kincaid Grade Level (FKGL) equates the readability of the text to the US school grade-level system required to comprehend the text. The equation underlying FKGL is given by:
  0.39×(words/sentences)+11.8×(syllables/words)−15.59
3. Gunning Fog Index (GFI) provides an estimation of the years of formal education required to comprehend text on the first reading. The target is to aim for a 7–8; if GFI is more than 12, the text is too difficult for most people to read. The equation underlying GFI is given by:
  0.4×([words/sentences]+100×[complexwords/words])
4. SMOG (Simple Measure of Gobbledygook) Index assesses the years of education one requires to comprehend writing. SMOG Index considers text for complex words containing three or more syllables.
  The equation underlying the SMOG Index is given by:
  1.0430×sqrt(30×complexwords/sentences)+3.1291
5. Coleman–Liau Index (CLI) is designed to assess the US grade level required to understand texts. It considers sentence length and word length to deduce readability. The equation underlying CLI is given by:
  5.89×(characters/words)−0.3×(sentences/words)−15.8
6. Automated Readability Index (ARI) determines how easy text is to understand and provides an estimate of the US grade level required to comprehend a passage. The equation underlying ARI is given by:
  4.71×(characters/words)+0.5×(words/sentences)−21.43

These selected tools comprise a blend of syllable-, word-, and sentence-based metrics capable of providing a robust quantitative analysis.

## Procedure

Each answer or information provided to the statements or questions on CMRI was copied into the readability online platform https://www.webfx.com/tools/read-able/ to calculate the scores for each of the above-listed indices. Non-text elements such as hyperlinks, tables, or images were excluded. Two reviewers conducted the readability assessments independently and achieved similar scores.

## Statistical analysis

Descriptive statistics were used to summarize readability scores across the five platforms. The mean, standard deviation (SD), and 95% confidence interval (CI) for each readability index were calculated. We checked for normal distribution for each group using Shapiro–Wilk. If each group is normally distributed, we performed one-way ANOVA. Homogeneity of variances was conducted using Levene’s test due to its robustness to violations of normality, which are commonly observed in real-world data. When ANOVA yielded significant results, Tukey’s Honest Significant Difference (HSD) test was used for *post hoc* comparisons to identify which groups differed significantly. Further, if normality was significantly violated in one or more groups, we used the non-parametric Kruskal–Wallis test instead. For significant Kruskal–Wallis results, we performed Dunn’s *post hoc* test to identify specific group differences. Additionally, multiple linear regression analysis was conducted to evaluate whether six text characteristics (i.e. number of sentences, total words, number of complex words, percentage of complex words, average words per sentence, and average syllables per word) significantly predicted FKRE readability scores for PEMs that exceeded recommended reading levels.

To assess the stability and practical significance of the identified predictors, a one-way sensitivity analysis was performed on the established multiple linear regression model for RadInfo and InsRad. Each of the six text statistics—Sentences, Words, Complex words, % Complex words, Average words per sentence, and Average syllables per word—was systematically varied across eight distinct, plausible values, while all other predictors were held constant at their respective baseline (average) values. The impact of these variations on the predicted FKRE score was subsequently recorded (see Supplementary data online, *Table S1*). A *P*-value ≤ 0.05 (two-tailed) was considered statistically significant.

## Results

**Figure 1:**
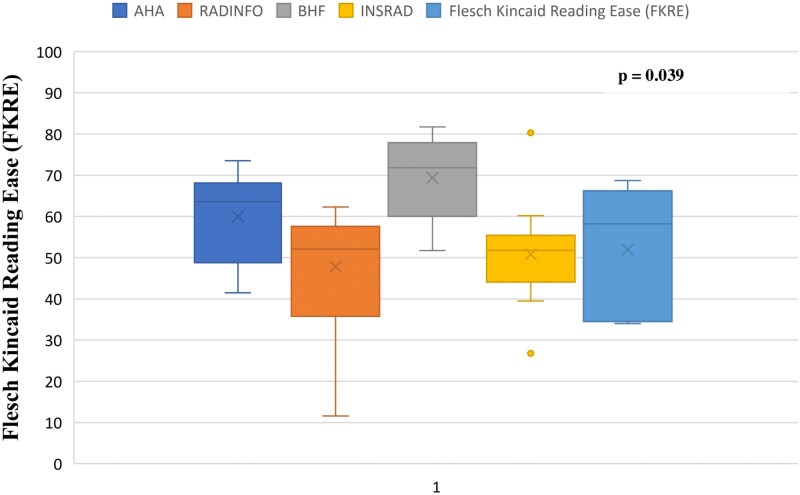
Readability of patient educational materials on CMRI from popular web portals.

*Figure 1* shows a graphical plot comparing the readability between the AHA, RadInfo, BHF, InsRad and EsCardio websites, demonstrating that using the FKRE tool, the BHF website yielded the highest score (69.4 ± 10.9; 95% CI: 57.9, 80.9) compared to the AHA website (60.0 ± 11.6; 95% CI: 47.8, 72.1), EsCardio (51.9 ± 16.3; 95% CI: 31.6, 72.2), InsRad (50.8 ± 13.3; 95% CI: 41.8, 59.8), and RadInfo (47.8 ± 15.3; 95% CI: 37.5, 58.1).

Comparing the readability of the patient education materials, BHF showed the highest average FKRE score, with an overall significance of *P* = 0.039 (*Figure 1*).

In *Table 1*, the CLI demonstrated a significant difference with RadInfo yielding the highest average score of 13.7 ± 2.5; 95% CI: 12.0, 15.4 compared to all the other websites (*P-*value = 0.009). Levene’s test showed no significant difference for the mean values (*P* = 0.541). No significant differences were observed for (Gunning Fog Score) GFS, SMOG, and ARI measures.

**Table 1:** Comparison of readability of patient educational materials on CMRI from popular web portals

Readability indices	AHA (mean ± SD; 95% CI)	RadInfo (mean ± SD; 95% CI)	BHF (mean ± SD; 95% CI)	InsRad (mean ± SD; 95% CI)	EsCardio (mean ± SD; 95% CI)	*P* value
FKRE	60.0 ± 11.6; 47.8, 72.1	47.8 ± 15.3; 37.5, 58.1	69.4 ± 10.9; 57.9, 80.9	50.8 ± 13.3; 41.8, 60.0	51.9 ± 16.3; 31.6, 72.2	0.039^a^
FKGL	9.2 ± 2.4; 6.6, 11.7	10.3 ± 2.3; 8.8, 11.9	8.2 ± 2.8; 5.3, 11.1	11.4 ± 2.7; 9.5, 13.2	10.4 ± 3.2; 6.5, 14.4	0.194^b^
GFS	12.1 ± 3.3; 8.7, 15.6	13.1 ± 2.3; 11.6, 14.7	11.3 ± 3.1; 8.0, 14.5	14.1 ± 3.1; 12.0, 16.2	13.5 ± 3.7; 8.9, 18.2	0.237^a^
SMOG	8.7 ± 2.4; 6.2, 11.2	9.6 ± 1.7; 8.5, 10.7	7.9 ± 2.1; 5.7, 10.0	10.1 ± 1.6; 9.0, 11.2	10.0 ± 2.7; 6.6, 13.3	0.183^a^
CLI	12.0 ± 1.8; 9: 10.0, 13.9	13.7 ± 2.5; 12.0, 15.4	9.8 ± 1.2; 8.5, 11.0	11.9 ± 1.8; 10.7, 13.1	12.4 ± 1.6; 10.4, 14.3	0.009^b^
ARI	9.4 ± 2.8; 6.5, 12.3	9.7 ± 2.4; 8.1, 11.3	8.4 ± 3.5; 4.7, 12.0	11.2 ± 3.3; 8.9, 13.4	10.0 ± 2.0; 7.5, 12.5	0.402^b^
Sentences	9.3 ± 7.3; 1.7, 17.0	20.8 ± 14.6; 11.0, 30.7	10.7 ± 8.1; 2.1, 19.2	10.9 ± 13.2; 2.1, 19.8	10.0 ± 8.3; −0.3, 20.3	0.163^a^
Words	140.0 ± 94.6; 40.7, 239.3	320.0 ± 233.7; 163.0, 477.0	200.0 ± 138.8; 54.0, 345.4	239.1 ± 283.9; 48.3, 429.8	161.6 ± 132.4; −2.8, 326	0.345^a^
Complex words	18.2 ± 13.5; 4.0, 32.4	57.6 ± 43.3; 28.5, 86.8	17.5 ± 11.5; 5.4, 29.6	35.0 ± 42.4; 6.5, 63.5	24.6 ± 17.9; 2.4, 46.8	0.134^a^
% of complex words	13.3 ± 5.3; 7.7, 18.8	18.3 ± 6.0; 14.2, 22.3	9.9 ± 3.3; 6.5, 13.3	15.1 ± 4.3; 12.2, 18.1	16.8 ± 5.8; 9.7, 24.0	0.033^b^
Average words per sentence	17.1 ± 4.1; 12.8, 21.3	15.1 ± 1.4; 14.1, 16.1	18.7 ± 5.3; 13.1, 24.3	20.9 ± 6.5; 16.6, 25.2	17.8 ± 4.6; 12.1, 23.5	0.106^a^
Average syllables per word	1.5 ± 0.1; 1.4, 1.7	1.7 ± 0.2; 1.6, 1.8	1.4 ± 0.1; 1.3, 1.5	1.6 ± 0.2; 1.5, 1.7	1.6 ± 0.2; 1.4, 1.8	0.005^a^

^a^Kruskal–Wallis test.

^b^One-way ANOVA test.

Note: Values presented as mean ± SD; 95% Confidence Interval.

*Readability Index Definitions and Interpretation Ranges: FKRE* scores range from 0 to 100 with higher scores indicating easier readability: 90–100 = Very easy (5th grade); 80–89 = Easy (6th grade); 70–79 = Fairly easy (7th grade); 60–69 = Standard (8th–9th grade); 50–59 = Fairly difficult (10th–12th grade); 30–49 = Difficult (College); 0–29 = Very confusing (College graduate).

*FKGL* (Flesch–Kincaid Grade Level): indicates the US school grade level required to understand the text.

*GFS* (Gunning Fog Score): grade level; a score of 12 implies senior high school level.

*SMOG* (Simple Measure of Gobbledygook): estimates years of education needed to understand the text.

*CLI* (Coleman–Liau Index): also grade-level based, using characters per word and sentence length.

*ARI (Automated Readability Index):* estimates the US grade level using character and sentence counts.

*Text Features—Complex words:* words with three or more syllables; *% Complex words*: proportion of complex words in the total word count; *Average words per sentence* and *average syllables per word* are indicators of sentence and word complexity.

Regarding the text statistics, overall, the PEMs on the RadInfo website had the highest average % of complex words compared to all the others (*P* = 0.033). The InsRad website demonstrated the highest average words per sentence (20.9 ± 6.5; 95%CI: 16.6, 25.2) than all the other; however, overall, this was not significant. On the other hand, RadInfo demonstrated the highest average syllables per word than those found on the other websites (*P* = 0.005). No significant differences were observed for words, complex words, and average words per sentence.

*Post-hoc* analysis was performed for results that showed significant differences in *Table 1* as follows: (1) Dunn’s test for FKRE which demonstrated a significant difference between the following: RadInfo vs. BHF (*P* = 0.001) and BHF vs. InsRad (*P* = 0.008); (2) Tukey HSD test for CLI which showed a significant difference between RadInfo vs. BHF (*P* = 0.003); (3) Tukey HSD test for % of complex words which showed a significant difference between RadInfo vs. BHF, with the average % of complex words higher on Radinfo website compared to the BHF website (*P* = 0.021), and (4) Dunn’s test for average syllables per word which revealed a significant difference between: RadInfo vs. BHF (*P* = 0.001), BHF vs. InsRad (*P* = 0.013), and BHF vs. EsCardio (*P* = 0.020).

Multiple linear regression was used to test if the 6 text statistics (i.e. sentences, words, complex words, % complex words, average words per sentence, and average syllables per word) predicted average FKRE scores of PEMs on websites whose information exceeded the recommended levels. While the RadInfo, InsRad, and EsCardio demonstrated FKRE scores that equate to ‘difficult to read,’ we only carried out the regression on RadInfo and InsRad separately. EsCardio was excluded because its PEM on CMRI consisted limited number of data points to meet statistical assumptions for regression modelling, which rendered it unsuitable for reliable estimation of predictor effects or detection of meaningful trends in the FKRE score.

For RadInfo (*Table 2*), the overall regression was statistically significant [R2 = 1.00, F(6, 4) = 6285.77, *P* = < 0.000]. The significant predictors of FKRE were average words per sentence (β = −1.23, *P* = < 0.000), and average syllables per word (β = 84.44, *P* = < 0.000).

**Table 2:** Multiple linear regression analysis predicting FKRE scores for PEMs on the RadInfo website

Predictor variables	Β	SE	t-stat	*P*-value	95% CI
Intercept	209.79	2.87	73.06	2.103E-07	201.82, 217.76
Sentences	−0.12	0.07	−1.85	0.138	−0.32, 0.06
Words	0.01	0.00	1.96	0.121	−0.00, 0.02
Complex words	−0.01	0.01	−0.03	0.978	−0.03, 0.03
% Complex words	−0.00	0.04	−0.04	0.972	−0.11, 0.10
Average words per sentence	−1.23	0.10	12.00	< 0.001*	−1.51, −0.94
Average syllables per word	−84.44	1.49	−56.54	< 0.001*	−88.58, −80.29

*P-value  *<*  *0.05*.

**Figure 2:**
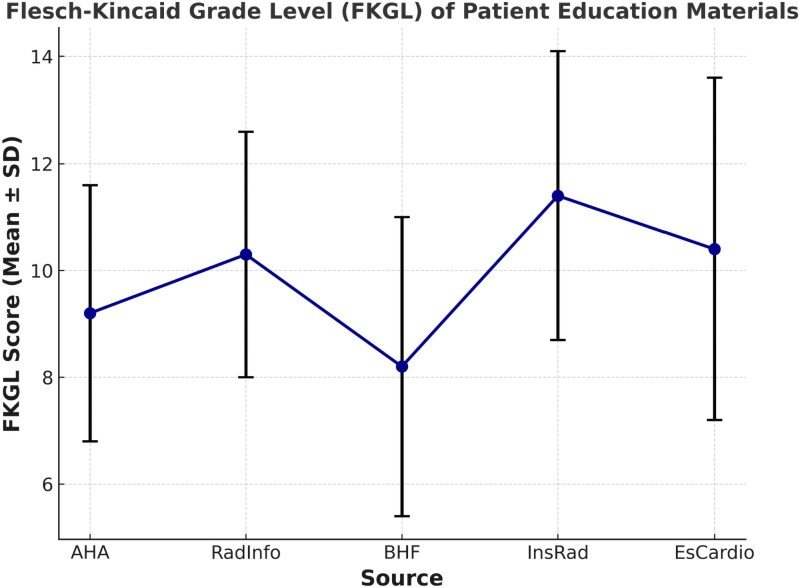
Comparison of average FKGL scores of patient educational materials on CMRI from popular web portals.

For InsRad (*Table 3*), the overall regression was also statistically significant [R2 = 1.00, F(6, 4) = 2035.01, *P* = < 0.001]. The significant predictors of FKRE were sentences (β = 0.90, *P* = 0.044), words (β = −0.06, *P* = 0.046), average words per sentence (β = −0.83, *P* = < 0.001), and average syllables per word (β = −85.0, *P* = < 0.001). In *Figure 2*, the BHF patient educational materials achieved the lowest FKGL score, while EsCardio had the highest score.

**Table 3:** Multiple Linear Regression Analysis Predicting FKRE Scores for PEMs on the InsRad website

Predictor variables	Β	SE	*t*-Stat	*P*-value	95% CI
Intercept	205.69	3.28	62.68	3.8807E-07	196.58, 214.80
Sentences	**0**.**90**	**0**.**31**	**2**.**91**	**0.044**	**0.04, 1.75**
Words	**−0**.**06**	**0**.**02**	**−2**.**85**	**0.046**	**−0.11, −0.00**
Complex words	0.12	0.05	2.38	0.076	−0.12, 0.25
% Complex words	−0.15	0.11	−1.28	0.268	−0.46, 0.17
Average words per sentence	**−0**.**83**	**0**.**07**	**−12**.**35**	< 0.001*	**−1.01, −0.64**
Average syllables per word	**−84**.**99**	**2**.**76**	**−30**.**84**	< 0.001*	**−92.65, −77.34**

*Statiscally significant boldened: *P-value*  *<*  *0.05*.

**Figure 3:**
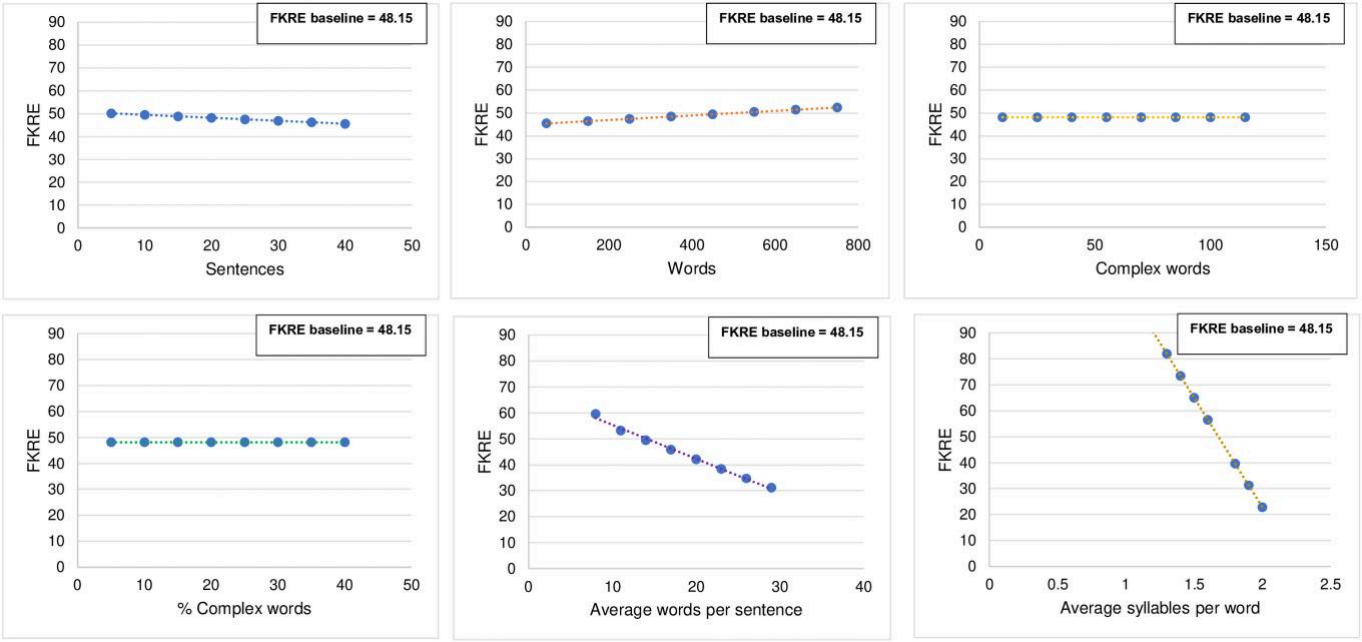
Sensitivity analysis: impact of text statistics on predicted FKRE for RadInfo.

The sensitivity analysis for the RadInfo multiple linear regression model uncovered a critical distinction in the influence of various text characteristics on the calculated FKRE score (*Figure 3*; Supplementary data online, *Table S1*). When *average words per sentence* and *average syllables per word* were varied, consistent, and discernible changes in the calculated FKRE were observed. In contrast, while subtle numerical fluctuations in FKRE were noted upon varying *sentences and words*, varying *complex words* and *% complex words* yielded no change in the calculated FKRE.

**Figure 4:**
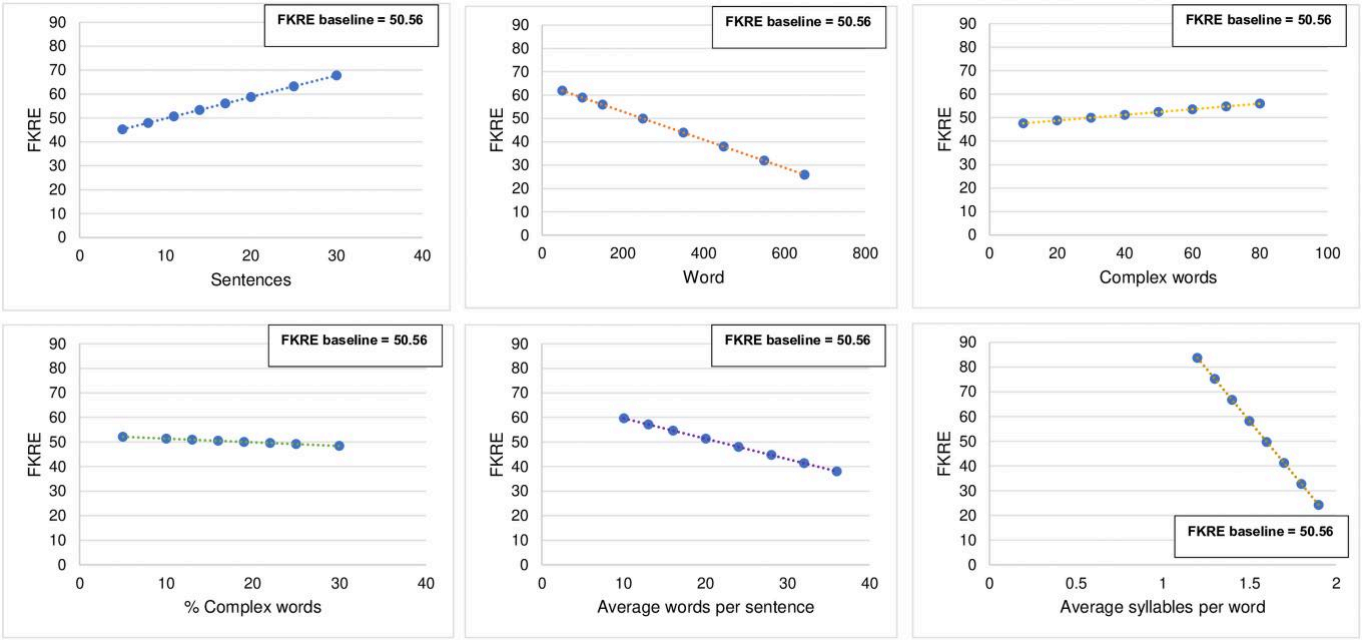
Sensitivity analysis: impact of text statistics on predicted FKRE for InsRad.

For the InsRad model, the sensitivity analysis revealed that varying all six text statistics technically led to changes in the calculated FKRE score (*Figure 4*; Supplementary data online, *Table S2*). However, the analysis clearly demonstrated that *average syllables per word* and *average words per sentence* were the dominant and most impactful drivers of FKRE variability. In contrast, while varying *sentences* and *words* also resulted in changes to FKRE, their respective *P*-values, though technically significant (*P* < 0.05), were notably closer to the significance threshold, and their coefficients were considerably smaller in magnitude than those of average word and sentence length. Furthermore, for *complex words* and *% complex words*, the observed FKRE changes were minimal.

## Discussion

To the best of our knowledge, there are no studies that have addressed the readability of web-based PEMs focused on CMRI. However, studies that have assessed the readability of web-based PEMs in imaging reported higher reading grade levels than recommended.^11,14,16^ Our study indicates that PEMs on CMRI from these popular web portals are written at higher reading grade levels than the recommended AMA and NIH levels. We noted that most websites’ PEMs were somewhat difficult to read for the public.

The average FKRE scores for both AHA and BHF equate to ‘standard (plain English), best comprehensible by 8th and 9th US grade levels.’ However, BHF demonstrated significantly better readability than both the RadInfo and InsRad. The average FKRE score for RadInfo equates to ‘difficult to read, requiring a college grade level’ to understand, while that of InsRad was ‘fairly difficult to read, requiring 10th- to 12th-grade (high school) level’. But EsCardio was also ‘fairly difficult to read and also requiring a 10th to 12th grade (high school) level’.

RadInfo website content had a significantly higher CLI, requiring a 14th reading grade level relative to that of BHF, which requires a 10th-grade reading level. Nonetheless, they both still exceed the recommended grade levels. Consistent with a previous study,^30^ though not imaging-related, we found no significant differences for GFS, SMOG, and ARI measures for all the websites; however, it is worth mentioning the interesting observations therein. The average GFS ranged from 11th- to 14th-grade level, but BHF yielded the lowest score at about 11th-grade level, still above the recommended grade levels. Further, we found the average SMOG to range from 8th- to 10th-grade level; however, BHF yielded the lowest score at almost 8th grade. Furthermore, although the ARI range from the 8th- to 10th-grade level, we found the EsCardio website yielded the highest score of 10th-grade level, while BHF again yielded the least at about the 8th-grade level.

Compared to all the others, the patient education material on the RadInfo website was difficult to read and had the highest average % of complex words and average syllables per word of all the other websites’ patient education materials. In addition, its average % of complex words and average syllables per word were significantly higher than those of the BHF. Importantly, the significant predictors of average FKRE on RadInfo include average words per sentence and average syllables per word. Indeed, when *average words per sentence* and *average syllables per word* were varied in the sensitivity analysis, consistent and discernible changes in the calculated FKRE were observed as opposed to varying others. This confirms their crucial role in dictating the comprehensibility of PEMs and highlighting that linguistic complexity, especially sentence structure and density of syllables, significantly reduced readability on this website. InsRad contents demonstrated ‘fairly difficult to read’, but we observed that the average syllables per word on both the InsRad and EsCardio websites were significantly higher than that of the BHF website. Compared to RadInfo, we found that more text statistics significantly predicted FKRE on InsRad, and this included sentences, words, average words per sentence, and average syllables per word. Though the sensitivity analysis for the InsRad model revealed that varying all six text statistics technically led to changes in the calculated FKRE score, the analysis clearly demonstrated that *average syllables per word* and *average words per sentence* were the dominant and most impactful drivers of FKRE variability. We highlight the critical importance of both lexical and syntactical simplicity in ensuring the comprehensibility of patient education materials.

Interestingly, the PEMs on the BHF website showed the lowest % of complex words and average syllables per word and demonstrated the most readable content of all the others. It requires an educational level of 8th and 9th grades to understand, equating to a standard (plain English).

Our findings of ‘difficult to read’ CMRI contents on the RadInfo, InsRad, and EsCardio websites are consistent with previous studies conducted on PEMs about imaging,^11,14,16^ and several other medical specialities.^30–35^ It is interesting to note that we sourced PEMs on CMRI from one of the websites (RadInfo) used in previous studies^14,16^ with similar quantitative readability scales, but the former also included four additional scales. Hansberry *et al*.^14^ first reviewed 138 online patient education articles on the website and noted that, on average, the articles were written for those between the 10th and 14th grades. In what seems to be an updated study to this, Bange *et al*.^16^ reported an average readability grade level to exceed the 11th-grade reading level for all the readability scales following their review of 131 patient education articles available in 2017 on the same website. None of the articles reviewed was found to be written at less than the 8th-grade or the 6th-grade levels. Despite the 5-year gap between the two studies, it appears that there has been no improvement in the readability of PEMs on the website. But then, pooling all patient education articles from the RadInfo website across diverse topics to obtain average readability elicits inherent limitations such as variability in word count and complexity, sentence structure, and terminology, which may mask readability concerns specific to niche medical topics, including CMRI. By contrast, we focused on a domain-specific analysis, which allowed for a targeted assessment of readability directed towards a specialized and complex description of CMRI.

Disadvantaged groups often have limited health literacy, and this includes those with language difficulties, cultural barriers, and people with conditions that affect comprehension (i.e. learning disability and dementia).^36^ Low health literacy has been associated with poor general health, increased hospital admissions, reduced use of preventative services, and reduced life expectancy.^37^ International multi-sector efforts have long recognised the essence of improving health literacy to reach a wide audience, including those accessing online information, and to provide an evidence-based health literacy environment, where health information is human-centred, accessible, culturally and linguistically appropriate, and supports life-long commitments to promote good health.^38–40^ Common health literacy strategies include avoiding technical jargon but using plain and direct language through careful application of good layout and design, and communicating with pictures, symbols, diagrams, videos, animations theatre performances.^38–40^ This approach has been reported to positively influence literacy levels^41,42^ but there is little evidence that it improves health outcomes.^38^

The findings hold significant implications for specific patient groups who are disproportionately affected by complex health information. For elderly individuals, who may experience age-related cognitive decline, vision impairments, or have less familiarity with digital health resources, overly technical or lengthy PEMs can severely impede their ability to understand critical medication instructions or health advice. Similarly, individuals with lower educational backgrounds are at a higher risk of limited health literacy, making them particularly vulnerable to information presented with high FKGL or complex terminology, potentially leading to medication non-adherence or poor self-management of chronic conditions. Finally, non-native English speakers face a double barrier, as they must not only navigate complex medical concepts but also process them in a non-primary language, further exacerbating comprehension difficulties.

## Limitations

There are limitations to our study. First, the various readability scales used in the present study have their inherent flaws. For example, the FKGL scale mainly considers sentence length, word syllable count, and does not consider visual or interactive content. Furthermore, we acknowledge that while formulas like the FKGL provide a general guide based on sentence length and word syllable count, they inherently do not account for crucial qualitative factors.

## Conclusion

Our study highlights a significant disparity in the readability of PEMs on CMRI across five major platforms. While resources from the AHA and BHF meet the recommended readability standards, materials hosted on RadInfo, InsRad, and the EsCardio websites fall into the ‘difficult to read’ or ‘fairly difficult to read’ categories. These findings underscore potential barriers to patient comprehension and informed decision-making. To improve accessibility of these websites, we recommend that these platforms revise their materials using plain language principles, such as reducing sentence length, limiting medical jargon, and replacing complex or multisyllabic words with simpler alternatives.

Future research should explore the real-world impact of PEM readability on patient comprehension, particularly through qualitative methodologies. In-depth interviews or focus groups could assess how patients from diverse backgrounds interpret and engage with PEMs on CMRI. Such insights would inform not only content revision but also guide the co-development of inclusive, patient-centred educational materials that promote health literacy and equitable care outcomes.

## Supplementary Material

qyaf111_Supplementary_Data

## Data Availability

The data that support the findings of this study are available from the corresponding author upon reasonable request.

## Lead author biography

Dr Albert D. Piersson is a senior lecturer and a course lead in Diagnostic Radiography & Medical Imaging at York St John University, UK. He has a multidisciplinary background spanning clinical radiography, cross-sectional imaging, sonography, academic leadership, and health technology research. He is committed to improving radiographic education, patient care, and workforce development. His research explores maternal and foetal health, global health inequalities, and the use of artificial intelligence in medical imaging. He is also a Senior Fellow of the Higher Education Academy and contributes actively to professional standards, curriculum innovation, and inclusive academic practice. He is a recipient of the Brain & Behavior Research Foundation (BBRF) Young Investigator Grant. He is currently involved in research projects related to foetal and early childhood neurodevelopmental trajectories.

## References

[1] World Health Organization . Cardiovascular disease. 2025. https://www.who.int/health-topics/cardiovascular-diseases#tab=tab_1 (2 February 2025 date last accessed).

[2] Salerno M, Sharif B, Arheden H, Kumar A, Axel L, Li D et al Recent advances in cardiovascular magnetic resonance: techniques and applications. Circ Cardiovasc Imaging 2017;10:e003951. 28611116 10.1161/CIRCIMAGING.116.003951 PMC5777859

[3] Ibrahim EH, Frank L, Baruah D, Arpinar VE, Nencka AS, Koch KM et al Value CMR: towards a comprehensive, rapid, cost-effective cardiovascular magnetic resonance imaging. Int J Biomed Imaging 2021;2021:8851958. 34054936 10.1155/2021/8851958 PMC8147553

[4] Nieto Alvarez I, Madl J, Becker L, Amft O. Patients’ experience to MRI examinations—a systematic qualitative review with meta-synthesis. J Magn Reson Imaging 2025;61:480–93. 38544326 10.1002/jmri.29365 PMC11645497

[5] Rooney MK, Santiago G, Perni S, Horowitz DP, McCall AR, Einstein AJ et al Readability of PEMsFrom high-impact medical journals: a 20-year analysis. J Patient Exp 2021;8:2374373521998847. 10.1177/2374373521998847 PMC8205335 34179407

[6] Weiss BD. Health literacy: a manual for clinicians. 2003. http://lib.ncfh.org/pdfs/6617.pdf (cited 2 January 2025).

[7] University of Oxford . The Passive and Active Voices and When to use Them: Oxford, UK: University of Oxford. [date unknown]. https://www.mpls.ox.ac.uk/training/resources-for-researcher-and-career-development/communication-skills/scientific-writing/the-passive-and-active-voices-and-when-to-to-use-them (2 March 2025 date last accessed).

[8] Literacy Project . Illiteracy by the numbers. 2022. https://literacyproj.org/ (2 March 2025 date last accessed).

[9] National Institutes of Health . Clear & simple: developing effective print materials for low-literate readers (NIH Publication No. 95-3594). 1994. https://www.nih.gov/institutes-nih/nih-office-director/office-communications-public-liaison/clear-communication/clear-simple (24 January 2025 date last accessed).

[10] Gov.UK . Content design: planning, writing and managing content. 2024. https://www.gov.uk/guidance/content-design/writing-for-gov-uk (5 February 2025 date last accessed).

[11] Delaney FT, Doinn TÓ, Broderick JM, Stanley E. Readability of PEMs related to radiation safety: what are the implications for patient-centred radiology care? Insights Imaging 2021;12:148. 34674063 10.1186/s13244-021-01094-3 PMC8531160

[12] Prabhu AV, Hansberry DR, Agarwal N, Clump DA, Heron DE. Radiation oncology and online patient education materials: deviating from NIH and AMA recommendations. Int J Radiat Oncol Biol Phys 2016;96:521–8. 27681748 10.1016/j.ijrobp.2016.06.2449

[13] Prabhu AV, Donovan AL, Crihalmeanu T, Hansberry DR, Agarwal N, Beriwal S et al Radiology online PEMs provided by major university hospitals: do they conform to NIH and AMA guidelines? Curr Probl Diagn Radiol 2018;47:75–9. 28669431 10.1067/j.cpradiol.2017.05.007

[14] Hansberry DR, John A, John E, Agarwal N, Gonzales SF, Baker SR. A critical review of the readability of online patient education resources from RadiologyInfo.org. Am J Roentgenol 2014;202:566–75. 24555593 10.2214/AJR.13.11223

[15] Hansberry DR, Shah K, Agarwal N, Kim SM, Intenzo CM. Nuclear medicine and resources for patients: how complex are online patient educational materials? J Nucl Med Technol 2018;46:144–6. 29438010 10.2967/jnmt.117.203380

[16] Bange M, Huh E, Novin SA, Hui FK, Yi PH. Readability of PEMsfrom RadiologyInfo.org: has there been progress over the past 5 years? Am J Roentgenol 2019;213:875–9. 31386570 10.2214/AJR.18.21047

[17] American Heart Association . Cardiac magnetic resonance imaging (MRI). 2025. https://www.heart.org/en/health-topics/heart-attack/diagnosing-a-heart-attack/magnetic-resonance-imaging-mri (12 Mar 2025 date last accessed).

[18] British Heart Foundation . CMRI scan. 2025 https://www.bhf.org.uk/informationsupport/tests/mri-scan (9 March 2025 date last accessed).

[19] Radiologyinfo.org . Cardiac (heart) MRI. 2025. https://www.radiologyinfo.org/en/info/cardiacmr (12 March 2025 date last accessed).

[20] InsideRadiology . MRI Heart: CMRI. 2025 .https://www.insideradiology.com.au/cardiac-mri/ (cited 2 April 2025).

[21] European Society of Cardiology . CMRI. 2025. https://www.escardio.org/static-file/Escardio/Subspecialty/EACVI/Advocacy/Cardiac%20MRI%20leaflet.pdf (10 March 2025 date last accessed).

[22] American Heart Association . About us. 2025. https://www.heart.org/en/about-us (9 March 2025 date last accessed).

[23] American Heart Association . Accessibility statement [Internet]. 2025. https://www.heart.org/en/about-us/statements-and-policies/accessibility-statement (12 March 2025 date last accessed)

[24] British Heart Foundation . Introduction: Help us fund the next breakthrough. 2025. https://www.bhf.org.uk/ (12 Mar 2025 date last accessed)

[25] World Wide Web Consortium (W3C). Web Content Accessibility Guidelines (WCAG) 2.0: Conformance. 2008a. https://www.w3.org/TR/WCAG20/#conformance (17 March 2025 date last accessed)

[26] World Wide Web Consortium (W3C) . Web Content Accessibility Guidelines (WCAG) 2.0. 2008b. https://www.w3.org/TR/WCAG20/ (15 March 2025 date last accessed).

[27] Radiologyinfo.org . Physician resources. 2025b. https://www.radiologyinfo.org/en/downloads#:∼:text=More%20than%2015.8%20Million%20Visits%20Over%20the%20Past%20Year (13 March 2025 date last accessed).

[28] InsideRadiology . Policies. 2025. https://www.insideradiology.com.au/policies/ (13 March 2025 date last accessed).

[29] European Society of Cardiology . A brief history of the ESC. 2024. https://www.escardio.org/The-ESC/About/A-brief-history-of-the-ESC (cited 13 March 2025).

[30] Wasir AS, Volgman AS, Jolly M. Assessing readability and comprehension of web-based PEMsby American Heart Association (AHA) and CardioSmart online platform by American College of Cardiology (ACC): how useful are these websites for patient understanding? Am Heart J Plus 2023;12:100308. 10.1016/j.ahjo.2023.100308 PMC10946022 38510202

[31] Sharma S, Latif Z, Makuvire TT, Taylor CN, Vargas F, Abo-Sido NS et al Readability and accessibility of patient-education materials for heart failure in the United States. J Card Fail 2025;31:154–7. 39094729 10.1016/j.cardfail.2024.06.015

[32] Kapoor K, George P, Evans MC, Miller WJ, Liu SS. Health literacy: readability of ACC/AHA online patient education material. Cardiol 2017;138:36–40. 10.1159/000475881 28571004

[33] Pashkova A, Bangalore R, Tan C, Svider PF, Korban A, Yam YK et al Assessing the readability of anesthesia-related PEMsfrom major anesthesiology organizations. Biomed Res Int 2022;2022:3284199. 35872854 10.1155/2022/3284199 PMC9300304

[34] Nash E, Bickerstaff M, Chetwynd AJ, Hawcutt DB, Oni L. The readability of parent information leaflets in paediatric studies. Pediatr Res 2023;94:1166–71. 37120650 10.1038/s41390-023-02608-z PMC10444605

[35] Williams AM, Muir KW, Rosdahl JA. Readability of PEMsin ophthalmology: a single-institution study and systematic review. BMC Ophthalmol 2016;16:133. 27487960 10.1186/s12886-016-0315-0 PMC4973096

[36] Gursul D . NIHR evidence: health information: are you getting your message across? NIHR Evid 2022.

[37] NHS . Health literacy. NHS Digital Service Manual; 2023. https://service-manual.nhs.uk/content/health-literacy (13 March 2025 date last accessed).

[38] Public Health England . Local Action on Health Inequalities: Improving Health Literacy to Reduce Health Inequalities. London: Public Health England; 2015. https://assets.publishing.service.gov.uk/government/uploads/system/uploads/attachment_data/file/460710/4b_Health_Literacy-Briefing.pdf (13 March 2025 date last accessed).

[39] US Centers for Disease Control and Prevention . National Action Plan to Improve Health Literacy. Atlanta: CDC; 2024 . https://www.cdc.gov/health-literacy/php/develop-plan/national-action-plan.html (24 March 2025 date last accessed).

[40] Department of Health and Aged Care . National Health Literacy Strategy Framework Consultation. Canberra: Department of Health and Aged Care; 2022 . https://consultations.health.gov.au/national-preventive-health-taskforce/national-health-literacy-strategy-framework-consul/ (13 Mar 2025 date last accessed).

[41] Clement S, Ibrahim S, Crichton N, Wolf M, Rowlands G. Complex interventions to improve the health of people with limited literacy: a systematic review. Patient Educ Couns 2009;75:340–51. 19261426 10.1016/j.pec.2009.01.008

[42] Meppelink CS, van Weert JC, Haven CJ, Smit EG. The effectiveness of health animations in audiences with different health literacy levels: an experimental study. J Med Internet Res 2015;17:e11. 25586711 10.2196/jmir.3979 PMC4319081

