# Supplementary material for: Readability of patient education materials on cardiac magnetic resonance imaging
Source: Eur Heart J Imaging Methods Pract. 2025 Aug 20;3(2):qyaf111. doi: 10.1093/ehjimp/qyaf111 (PMC12448386; doi:10.1093/ehjimp/qyaf111)
Supplement: qyaf111_Supplementary_Data [file qyaf111_supplementary_data.zip › Supplementary Table 1 .docx]

**Supplementary Table 1 Sensitivity Analysis Summary for RadInfo**

| **Sentences** | | |
| --- | --- | --- |
| **Input value of predictor** | **Value varied** | **Resulting FKRE** |
| 20.82 (Baseline) | NA | 48.15 |
|  | 5 | 50.21 |
|  | 10 | 49.56 |
|  | 15 | 48.91 |
|  | 20 | 48.26 |
|  | 25 | 47.61 |
|  | 30 | 46.96 |
|  | 35 | 46.31 |
|  | 40 | 45.66 |
| **Words** | | |
| 320 (Baseline) | NA | 48.15 |
|  | 50 | 45.45 |
|  | 150 | 46.45 |
|  | 250 | 47.45 |
|  | 350 | 48.45 |
|  | 450 | 49.45 |
|  | 550 | 50.45 |
|  | 650 | 51.45 |
|  | 750 | 52.45 |
| **Complex words** | | |
| 57.64 (Baseline) | NA | 48.15 |
|  | 10 | 48.15 |
|  | 25 | 48.15 |
|  | 40 | 48.15 |
|  | 55 | 48.15 |
|  | 70 | 48.15 |
|  | 85 | 48.15 |
|  | 100 | 48.15 |
|  | 115 | 48.15 |
| **% Complex words** | | |
| 18.27 (Baseline) | NA | 48.15 |
|  | 5 | 48.15 |
|  | 10 | 48.15 |
|  | 15 | 48.15 |
|  | 20 | 48.15 |
|  | 25 | 48.15 |
|  | 30 | 48.15 |
|  | 35 | 48.15 |
|  | 40 | 48.15 |
| **Average words per sentence** | | |
| 15.11 (Baseline) | NA | 48.15 |
|  | 8 | 59.60 |
|  | 11 | 53.21 |
|  | 14 | 49.52 |
|  | 17 | 45.83 |
|  | 20 | 42.14 |
|  | 23 | 38.45 |
|  | 26 | 34.76 |
|  | 29 | 31.07 |
| **Average syllables per word** | | |
| 1.7 (Baseline) | NA | 50.56 |
|  | 1.2 | 90.37 |
|  | 1.3 | 81.93 |
|  | 1.4 | 73.48 |
|  | 1.5 | 65.04 |
|  | 1.6 | 56.59 |
|  | 1.8 | 39.71 |
|  | 1.9 | 31.27 |
|  | 2.0 | 22.82 |
