# Supplementary material for: Readability of patient education materials on cardiac magnetic resonance imaging
Source: Eur Heart J Imaging Methods Pract. 2025 Aug 20;3(2):qyaf111. doi: 10.1093/ehjimp/qyaf111 (PMC12448386; doi:10.1093/ehjimp/qyaf111)
Supplement: qyaf111_Supplementary_Data [file qyaf111_supplementary_data.zip › Supplementary Table 2 .docx]

**Supplementary Table Sensitivity Analysis Summary for InsRad**

| **Sentences** | | |
| --- | --- | --- |
| **Input value of predictor** | **Value varied** | **Resulting FKRE** |
| 10.91 (Baseline) | NA | 50.56 |
|  | 5 | 45.25 |
|  | 8 | 47.95 |
|  | 11 | 50.65 |
|  | 14 | 53.35 |
|  | 17 | 56.05 |
|  | 20 | 58.75 |
|  | 25 | 63.25 |
|  | 30 | 67.75 |
| **Words** | | |
| 239.09 (Baseline) | NA | 50.56 |
|  | 50 | 61.91 |
|  | 100 | 58.91 |
|  | 150 | 55.91 |
|  | 250 | 49.91 |
|  | 350 | 43.91 |
|  | 450 | 37.91 |
|  | 550 | 31.91 |
|  | 650 | 25.91 |
| **Complex words** | | |
| 35 (Baseline) | NA | 50.56 |
|  | 10 | 47.56 |
|  | 20 | 48.76 |
|  | 30 | 49.96 |
|  | 40 | 51.16 |
|  | 50 | 52.36 |
|  | 60 | 53.56 |
|  | 70 | 54.76 |
|  | 80 | 55.96 |
| **% Complex words** | | |
| 15.4 (Baseline) | NA | 50.56 |
|  | 5 | 52.12 |
|  | 10 | 51.37 |
|  | 13 | 50.92 |
|  | 16 | 50.47 |
|  | 19 | 50.02 |
|  | 22 | 49.57 |
|  | 25 | 49.12 |
|  | 30 | 48.37 |
| **Average words per sentence** | | |
| 20.91 (Baseline) | NA | 50.56 |
|  | 10 | 59.62 |
|  | 13 | 57.13 |
|  | 16 | 54.64 |
|  | 20 | 51.32 |
|  | 24 | 48.00 |
|  | 28 | 44.68 |
|  | 32 | 41.36 |
|  | 36 | 38.04 |
| **Average syllables per word** | | |
| 1.59 (Baseline) | NA | 50.56 |
|  | 1.2 | 83.71 |
|  | 1.3 | 75.21 |
|  | 1.4 | 66.71 |
|  | 1.5 | 58.21 |
|  | 1.6 | 49.71 |
|  | 1.7 | 41.22 |
|  | 1.8 | 32.72 |
|  | 1.9 | 24.22 |
